# Acupuncture Enhances the Synaptic Dopamine Availability to Improve Motor Function in a Mouse Model of Parkinson's Disease

**DOI:** 10.1371/journal.pone.0027566

**Published:** 2011-11-22

**Authors:** Seung-Nam Kim, Ah-Reum Doo, Ji-Yeun Park, Hyungjin Bae, Younbyoung Chae, Insop Shim, Hyangsook Lee, Woongjoon Moon, Hyejung Lee, Hi-Joon Park

**Affiliations:** 1 Studies of Translational Acupuncture Research, Acupuncture and Meridian Science Research Center, Kyung Hee University, Seoul, Republic of Korea; 2 Department of Oriental Medical Science, Kyung Hee University, Seoul, Republic of Korea; National Institute of Health, United States of America

## Abstract

Parkinson's disease (PD) is caused by the selective loss of dopaminergic neurons in the substantia nigra (SN) and the depletion of striatal dopamine (DA). Acupuncture, as an alternative therapy for PD, has beneficial effects in both PD patients and PD animal models, although the underlying mechanisms therein remain uncertain. The present study investigated whether acupuncture treatment affected dopamine neurotransmission in a PD mouse model using 1-methyl-4-phenyl-1,2,3,6-tetrahydropyridine (MPTP). We found that acupuncture treatment at acupoint GB34 improved motor function with accompanying dopaminergic neuron protection against MPTP but did not restore striatal dopamine depletion. Instead, acupuncture treatment increased dopamine release that in turn, may lead to the enhancement of dopamine availability in the synaptic cleft. Moreover, acupuncture treatment mitigated MPTP-induced abnormal postsynaptic changes, suggesting that acupuncture treatment may increase postsynaptic dopamine neurotransmission and facilitate the normalization of basal ganglia activity. These results suggest that the acupuncture-induced enhancement of synaptic dopamine availability may play a critical role in motor function improvement against MPTP.

## Introduction

Dopaminergic system dysfunction is implicated in a wide variety of neurological disorders, including Parkinson's disease (PD). PD is characterized by the selective loss of dopaminergic neurons in the substantia nigra (SN) and a depletion of striatal dopamine (DA), and dopamine depletion in PD leads to abnormal changes of basal ganglia activity that in turn, result in an inability to control voluntary movement [1]. Dopamine replacement therapies remain the most effective clinical option for PD patients despite the occasionally severe side effects [2].

Recent studies from several laboratories including ours have shown that acupuncture has a beneficial effect in rodent models of PD [3], [4], [5]. In both the 6-hydroxydopamine (6-OHDA) lesioned rat and 1-methyl-4-phenyl-1,2,3,6-tetrahydropyridine (MPTP) lesioned mouse, acupuncture has proven to be neuroprotective. We have demonstrated that acupuncture rescues dopaminergic neurons by increasing the expressions of trkB [5], cycolphilin A [3], and Akt [6] and by decreasing the inflammation in the substantia nigra [7]. Acupuncture has also been shown to reduce the oxidative stress in the substantia nigra and striatum [8], [9]. However, the underlying mechanisms of acupuncture on the improvement of motor dysfunction in PD models are not well understood.

Given the critical role of dopamine and the importance in the neuroplasticity of the impaired basal ganglia on regulating motor function in PD, we hypothesized that acupuncture improves the motor deficits by modulating dopaminergic neurotransmission in the striatum, and thus ameliorating the abnormal postsynaptic changes induced by dopamine depletion in mouse Parkinsonian model. Thus, in the present study, we compared the effects of acupuncture on the motor function and dopaminergic neuron survival with sham acupuncture (the same acupuncture stimulation was given to a control point) in mouse Parkinsonian model. The changes in the expressions of the phosphorylated DARPP-32 and FosB as well as the dopamine contents, dopamine efflux, and turnover ratios were measured to investigate the role of acupuncture against the changes induced by dopamine depletion in the striatum.

## Materials and Methods

### Animals and MPTP intoxication

**Figure 1:**
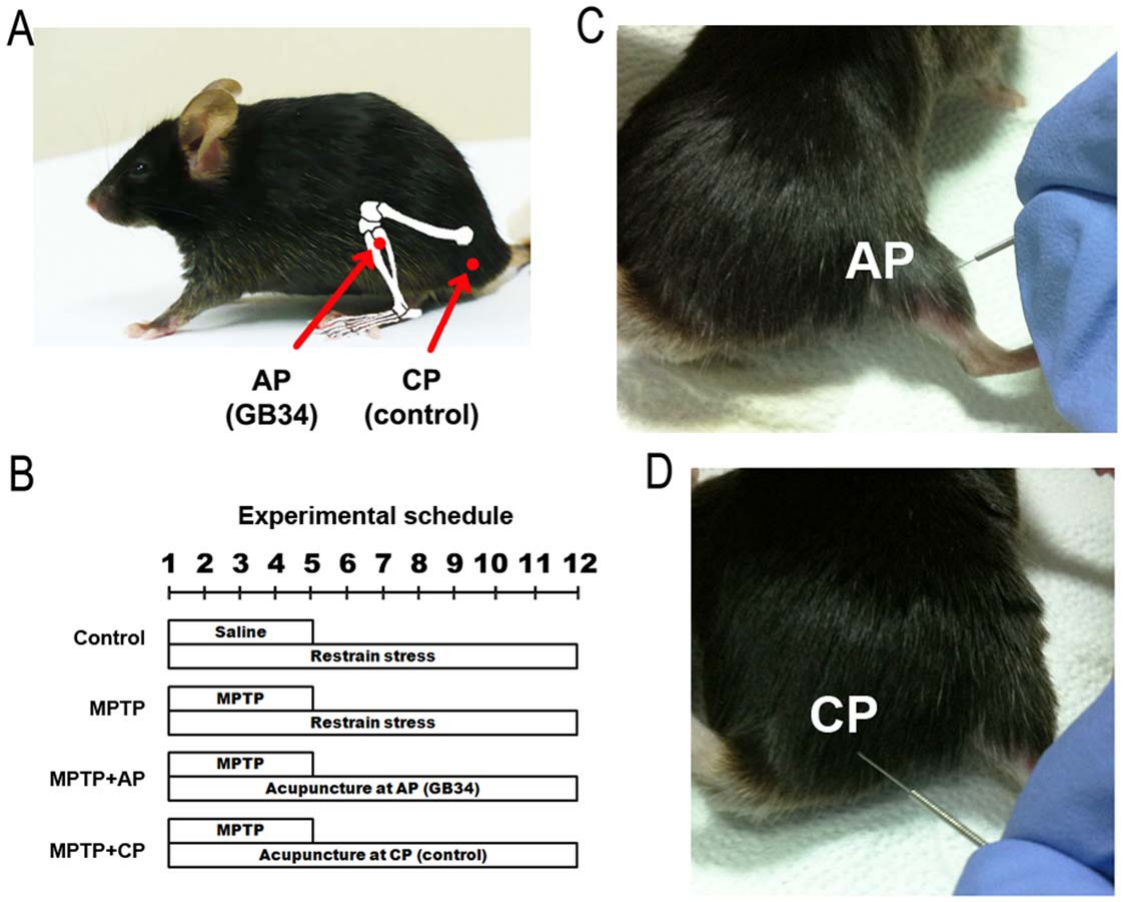
Acupuncture treatment and experimental procedure. (A) The point location of acupuncture treatment (red circle). ‘AP (GB34)’ refers to acupoint GB34. ‘CP’ (control point) refers to control point. (B) Experimental schedule. Mice in the MPTP, MPTP+AP, and MPTP+CP groups were treated with 30 mg/kg of MPTP for five consecutive days, unless Control group were treated with saline instead of MPTP. Acupuncture treatment was performed at acupoint GB34 (MPTP+AP) or a control point (MPTP+CP) once a day for 12 consecutive days after the first MPTP administration. (C, D) Photographs of acupuncture treatment at GB34 (C) and control point (D). The mice were immobilised by holding their neck. Acupuncture needles were inserted, turned at a rate of two spins per second for 15 seconds, and then immediately removed.

All experiments were approved by the Kyung Hee University Animal Care Committee for animal welfare [KHUASP(SE)-09-046] and were maintained in strict accordance with Guidelines of the NIH and Korean Academy of Medical Sciences. Twelve-week-old male C57BL/6 mice (Central Lab. Animal Inc., Seoul, Republic of Korea), weighing 23–26 g each, were used in all of the experiments. The mice were divided into Control, MPTP (MPTP only), MPTP with acupuncture treatment at acupoint GB34 (MPTP+AP), and MPTP with sham acupuncture at a control point (MPTP+CP) groups. The mice in all of the MPTP groups (MPTP, MPTP+AP, and MPTP+CP groups) received an intraperitoneal injection of MPTP-HCl (30 mg/kg of free base; Sigma-Aldrich, St. Louis, MO, USA) in saline at 24-h intervals for five consecutive days. The mice in the Control group were injected with saline instead of MPTP (Fig. 1B).

### Acupuncture treatment

Two hours after each MPTP injection, acupuncture stimulation was performed at an acupoint GB34 (MPTP+AP group) or at a control point (MPTP+CP group) (Fig. 1A). After the final MPTP injection, acupuncture stimulation continued daily for seven days (total 12 days). In order to rule out the non-specific effects of acupuncture, we used the sham acupuncture group in which the same acupuncture stimulation was given to the control point. Acupoint GB34 has been used to treat movement disorders in traditional East Asian medicine [10], and also studied on the relation with motor function in recent neuroimaging studies [11], [12]. Since we previously found that acupoint GB34 exerted the highest neuroprotective effect among several candidate acupoints (GB34, SI3, BL62, and ST36) using the same MPTP protocol [3], GB34 was chosen for the present study. It is located at the point of the intersection of lines from the anterior border to the head of the fibula [13], [14]. The control point was set at a point that was approximately 3 mm to the lateral side of the tail on the gluteus muscle (Fig. 1C, D). The mice were lightly immobilised to minimize the restraint stress. Acupuncture needles (15 mm in length, 0.20 mm in diameter; Haeng-lim-seo-weon Acuneedle Co., Seoul, Republic of Korea) were bilaterally inserted to a depth of 3 mm at GB34 or the control point, turned at a rate of two spins per second for 15 seconds, and then immediately removed. All mice in all groups were also gently immobilized by holding their necks with the head in an upright position for 15 seconds, to give the same immobilization stress as the acupuncture group.

### Behavioural test

A rotarod instrument (MED associates Inc., St. Albans, VT, USA) was used to simultaneously record the latency to fall of five mice. The overall rod performance (ORP) method was used, as previously described [15]. On day seven after the last MPTP or saline injection, the mice were pre-trained three times with 1-h intervals on an accelerating rod speed mode. The time on the rod was recorded with a maximum of 240 seconds for successive rotational speeds (16, 20, 24, 28, and 32 rpm), and the ORP score was calculated by the trapezoidal method [16]. All assessors were blinded to the expected results and experimental groups.

### Immunohistochemistry

On day seven after the last MPTP or saline injection, the mice were transcardially perfused with 4% paraformaldehyde in a 0.2 M phosphate buffer. Free-floating sections encompassing the entire midbrain and striatum (40 µm thickness). Tissues, which are between AP −3.08∼−3.28 mm from bregma for midbrain and between AP +0.38∼+0.98 mm from bregma for striatum in accordance with the atlas of the mouse brain [17], were selected for analysis. The selected tissues were removed, fixed, and cryoprotected. An immunohistochemistry method was performed, as previously described [18], [19]. After incubation with 3% H_2_O_2_ in a 0.05 M phosphate-buffered saline, the sections were blocked with 1% BSA and normal goat serum. The sections were incubated overnight at room temperature in primary antibodies: rabbit tyrosine hydroxylase (TH, 1∶1,000; Santa Cruz Biotechnology, Santa Cruz, CA, USA), dopamine transporter (DAT, 1∶500; Millipore Co., Billerica, MA, USA), Thr-34-phospho-DARPP-32 (1∶500; Abcam Inc., Cambridge, MA, USA), and FosB (1∶500; Cell Signalling Technology, Danvers, MA, USA). The tissue sections were incubated with biotinylated anti-rabbit IgG (Vector Laboratories, Inc., Burlingame, CA, USA) for 1 h at room temperature, incubated with ABC reagent (Vector Laboratories Inc., Burlingame, CA, USA) for 1 h at room temperature, and incubated for 2 min in 0.02% diaminobenzidine and 0.003% hydrogen peroxide in 1 M Tris-buffered saline (pH 7.5). After the reaction, the tissue sections were mounted on gelatin-coated slides, dried, dehydrated, and covered. For Nissl double staining, 0.5% Cresyl violet solution was used for 5 min before mounting.

### Image analysis

For stereological analysis of dopaminergic neuron cell counting, TH- and Nissl-positive cells in the substantia nigra pars compacta (SNpc) were counted bilaterally in five continuous 40 µm thickness immunostained mesencephalic sections at the level between AP −3.08∼−3.28 mm from bregma (accordance with atlas of the mice brain) using a Zeiss Axioscope 2 (Zeiss, Jena, Germany) and an attached StereoInvestigator system (MicroBrightfield, VT, USA). Stereological methods were used to analyze total volume of the SNpc using optical fractionator (StereoInvestigator system; MicroBrightfield, VT, USA). After counting, the SNpc nuclei were reconstructed serially and their volume calculated with StereoInvestigator software. The optical fractionator stereological probe was used to determine neuronal count in the SNpc in sections stained for both Nissl and TH. For dopaminergic fiber analysis, pictures of the striatum were taken using a bright-field microscope (BX51; Olympus, Tokyo, Japan). Immunoreactive optical densities were calculated using Image Pro software (version 6.0 for Windows; Media Cybernetics Inc., Bethesda, MD, USA). To correct for background differences, the optical density of the corpus callosum of each brain tissue was selected and used for normalization. p-DARPP-32 and FosB counts were performed by sampling a 500 µm wide by 350 µm high area where 1 mm distant from the vertical center of the brain and under the corpus callosum.

### In vivo microdialysis

The mice were anaesthetised with an anaesthetic solution containing tiletamine/zolazepam 30 mg/kg (Zoletile; Virbac, France) and xylazine 10 mg/kg (Rompun; Bayer, Korea). An intracerebral guide cannula (MBR-5; BASi, USA) was stereotaxically implanted into the striatum (AP +0.1; ML +0.2; DV −0.2 from bregma), and coordinates are in accordance with atlas of the mice brain. Acrylic cement (GC Corporation) was used for fixation of the guide cannulas. Then the mice were placed individual home cage 24 h for recovery from surgery. On the day of the experiment, a microdialysis probe (MBR-2–5, 0.24 mm diameter, 2 mm membrane length, cut-off 38 KDa; BASi, USA) was inserted through the guide cannula. The probes were connected to a microinjection syringe pump (CMA/Microdialysis) and filtered Ringer's solution (145 mM NaCl, 3 mM KCl, 1 mM MgCl_2_, 1.2 mM CaCl_2_, pH 7.4) perfused the probes at a flow rate of 1 µl/min. After insertion of the probes, samples were collected during 20-min throughout the experimental period. And total 220-min samples were collected. The experiment was performed under normal light conditions in an undisturbed room. The dialysates were stored at −80°C until DA determination with high-performance liquid chromatography (HPLC).

### High performance liquid chromatography (HPLC) analysis

The tissue contents of the neurotransmitters were measured using HPLC (Waters Co., Milford, MA, USA) in combination with an electrochemical detecting system (ESA Coulochem III detection system, range 100 nA, potential −100 mV to +320 mV; ESA Inc., Chelmsford, MA, USA). Mice were sacrificed on day seven after the last MPTP or saline injection, and the striatum of each mouse was rapidly dissected, frozen in liquid nitrogen, and stored at −72°C until assayed. These brain striatum tissues were homogenized in 300 µl of 0.1% perchloric acid. The resultant homogenates were centrifuged for 20 minutes at 1000× g at 4°C. The supernatant was filtered through a 0.22 µm membrane, and an aliquot (15 µl in volume) of the resulting solution was injected into the HPLC pump. Chromatographic separation was performed using a C18 reverse-phase column (150 mm×3.9 mm, 4 µm; Waters Co., Milford, MA, USA), and data analyses were performed using computer software (Empower; Waters Co., Milford, MA, USA). The mobile phase, which had a pH of 3.2, consisted of 150 mM sodium phosphate monobasic monohydrate, 1.85 mM octanesulfonic acid, 0.05 mM EDTA, 0.01% triethylamine, 4% methanol, and 6% acetonitrile. DA, 3,4, dihydroxyphenylacetic acid (DOPAC), homovanillic acid (HVA), and serotonin standards were prepared in 0.1% perchloric acid (Sigma-Aldrich, St. Louis, MO, USA). Each concentration was adjusted with respect to the standard and quantified from a standard curve. The levels of DA, DOPAC, HVA, and serotonin were calculated as nanograms per microgram of total protein.

### Data analysis

All procedures, assessments, and analyses were performed in a blinded manner to minimise the observer bias. The SPSS, version 17.0 (SPSS Inc., Chicago, IL, USA), was used for statistical procedures. All data are expressed as the mean ± SEM. A statistical analysis was performed using one-way ANOVA followed by a post-hoc Newman-Keuls test. The effect of acupuncture stimulation on dopamine efflux was determined by two-way ANOVA analysis with repeated measures over time followed by Bonferroni's post-hoc test. In all of the analyses, differences were considered to be statistically significant at *P*<0.05.

## Results

### Acupuncture treatment at GB34 improved motor function and prevented dopaminergic neuron degeneration against MPTP

**Figure 2:**
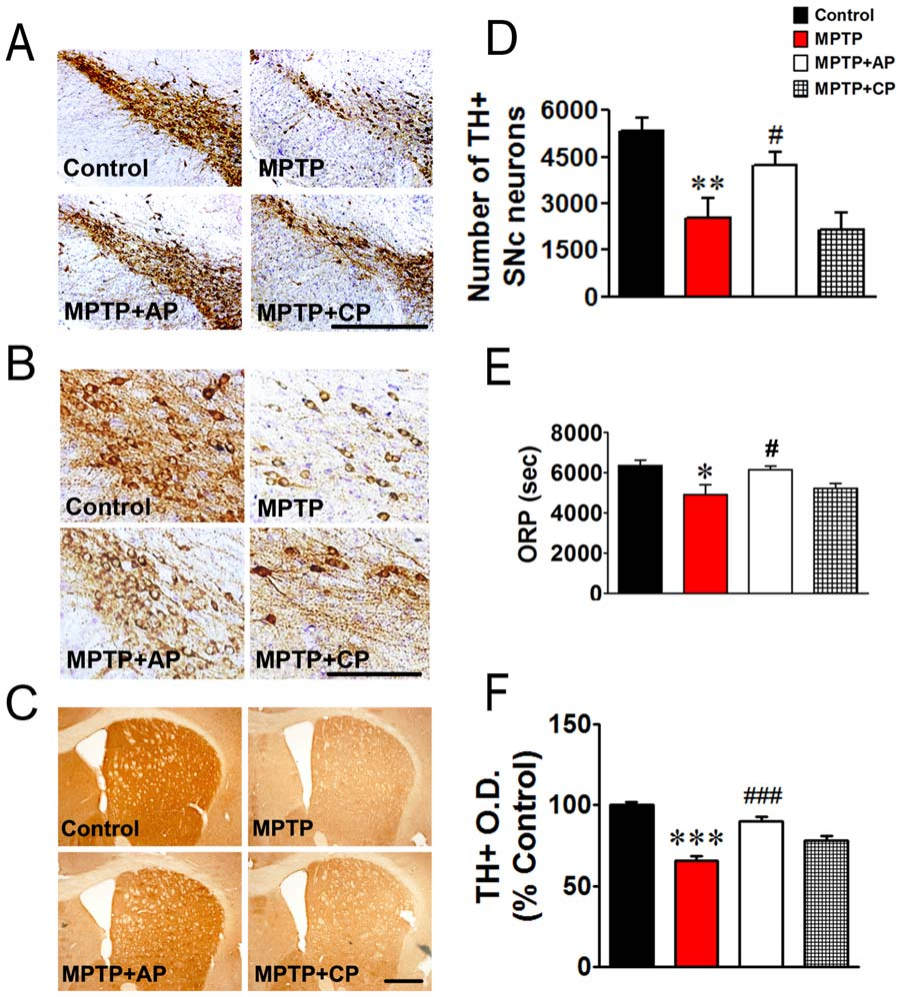
The behavioural and neuroprotective effects of acupuncture treatment at GB34 in MPTP mice. (A, B) Immunohistochemical staining for TH-positive and Nissl-positive dopaminergic neurons in the substantia nigra in a low magnification (A, scale bar: 300 µm) and in a higher magnification (B, scale bar: 100 µm). (C) Immunohistochemical staining for TH-positive dopaminergic fibers in the striatum. (Scale bar: 500 µm.) (D) Bar graph of TH-positive cell counts in the substantia nigra of each group. (E) Bar graph of the overall rod performance (ORP) scores of each group. (F) Bar graph of optical density of TH-positive fibers in the striatum of each group (n = 7–10 per group). A significant recovery of TH-positive cell numbers and fibers in the MPTP+AP group was observed. Also, acupuncture treatment at GB34 significantly improved motor function against MPTP. Data are normalized to the Control group. *P<0.05, **P<0.01 and ***P<0.001 versus Control group, #*P*<0.05 and ###*P*<0.001 versus MPTP group via one-way ANOVA followed by a Newman-Keuls test.

Previously, we reported that GB34 exerted the highest neuroprotective effect among several candidate acupoints in a mice Parkinsonian model [3], [8]. In this study, we first examined whether this acupoint was indeed critical for the effects of acupuncture against MPTP. Acupuncture treatment was performed at GB34 (MPTP+AP group) or the control point (MPTP+CP group) once a day for 12 consecutive days after the first MPTP administration (Fig. 1). Acupuncture treatment at GB34 induced a significant improvement of motor function against MPTP, whereas acupuncture treatment at the control point had no effect. Mice in the MPTP group showed significantly lower overall rod performance (ORP) scores (4901.3±501.9) in comparison to mice in the Control group (6347.7±272.2, *P*<0.05). Mice in the MPTP+AP group showed significantly higher ORP scores (6148.7±178.0) in comparison to mice in the MPTP group (*P*<0.05). The ORP scores were not statistically different between the MPTP and MPTP+CP groups (5217.7±251.6, Fig. 2E).

Also, a significant recovery of TH-positive cell numbers in the MPTP+AP group was observed. The MPTP group had significantly fewer TH-positive neurons in the SN (2536.83±626.06) in comparison to those observed in the Control group (5309.90±461.48, *P*<0.01); however, the MPTP+AP group had significantly more TH-positive neurons in the SN (4233.34±405.72) in comparison to those observed in the MPTP group (*P*<0.05). There was no statistical difference between the MPTP and the MPTP+CP groups (2140.07±543.33, Figs. 2A, B, D). To further support TH-positive cell count result, we also performed Nissl-positive cell counts (supplementary figure S2). Consistent with TH-positive cell counts, MPTP caused a considerable loss of Nissl-positive cells in the substantia nigra pars compacta (SNc), while acupuncture treatment led to a significant recovery of Nissl-positive cell numbers in the SNc against MPTP. In the striatum, acupuncture treatment also showed similar protection of dopaminergic fibers against MPTP toxicity (65.61±2.85% vs. 90.14±2.47% of Control group, *P*<0.001, Figs. 2C, F). These results demonstrate that acupuncture treatment at a specific acupoint (GB34), but not at a control point, significantly alleviated MPTP-induced motor dysfunction and dopaminergic neuron degeneration.

Since all of our above results display that acupuncture on control point did not produce any effects, we did not use the MPTP+CP group in the following experiments to minimize the number of animals.

### Acupuncture treatment at GB34 increased dopamine turnover ratios in the striatum

**Figure 3:**
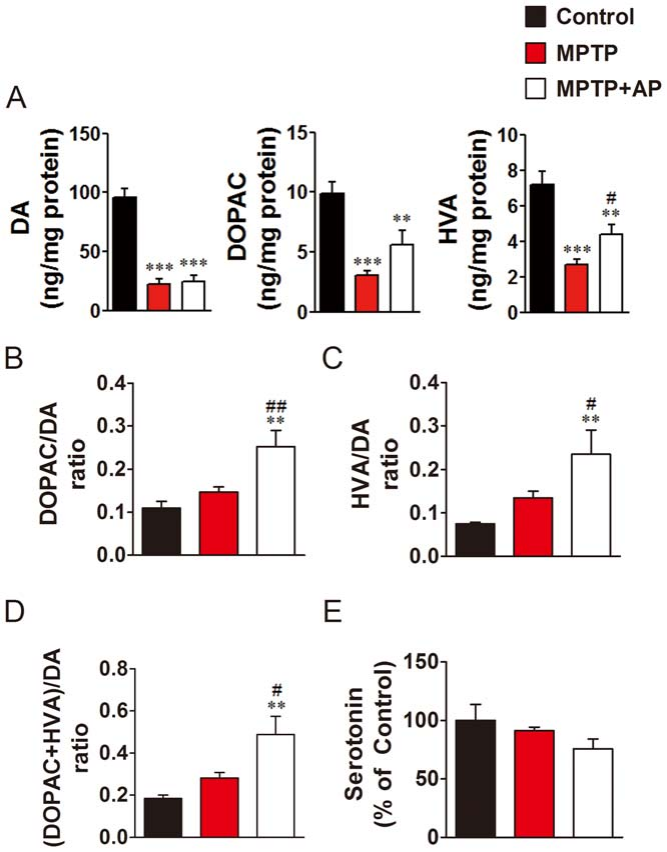
Tissue dopamine contents and its turnover ratios in the striatum of each group. (A) Contents of dopamine and its metabolates, DOPAC and HVA, in the striatum of each group (n = 8 per group). (B) DOPAC/DA, (C) HVA/DA, and (D) (DOPAC plus HVA)/DA turnover ratio of each group (n = 8 per group). (E) Serotonin levels of each group (n = 8 per group). MPTP administration induced a significant decrease in the dopamine and its metabolates levels, whereas acupuncture treatment at GB34 significantly increased the dopamine turnover ratio. **P<0.01 and ***P<0.001 compared to Control, #P<0.05 and ##P<0.01 compared to MPTP group, one-way ANOVA, followed by Newman-Keuls test.

To examine any possible effect of acupuncture treatment on the dopaminergic system, the levels of striatal DA and its metabolites, DOPAC and HVA, were measured using HPLC. In this analysis, the levels of serotonin in the same brain were measured for internal control. In Figure 3, HPLC analysis revealed that MPTP administration induced a significant 80% depletion in striatal dopamine levels in both the MPTP and MPTP+AP groups in comparison to the Control group (95.7±10.5 vs. 22.1±4.4 ng/mg protein, *P*<0.001, Fig. 3A), whereas serotonin levels were not statistically different among the groups (43.0±6.0 vs. 39.3±1.3 ng/mg protein, *P*>0.05, Fig. 3E). There were no significant differences in striatal dopamine levels between the MPTP and MPTP+AP groups (22.1±4.4 vs. 24.4±4.7 ng/mg protein, Fig. 3A); however, there was a significant increase in HVA in the MPTP+AP group in comparison to that observed in the MPTP group (2.7±0.3 vs. 4.4±0.5 ng/mg protein, *P*<0.05, Fig. 3A). Moreover, there were significant increases in dopamine turnover ratios (DOPAC/DA, HVA/DA, and DOPAC+HVA/DA) in the MPTP+AP group (25.3±3.5, 23.6±5.1, and 48.9±8.1) in comparison to both the MPTP (14.7±1.2, *P*<0.01; 13.5±1.5 and 28.2±2.6, *P*<0.05 each) and Control groups (10.9±2.2, 7.5±0.5, and 18.4±2.3, *P*<0.01 each, Figs. 3B, C, D).

**Figure 4:**
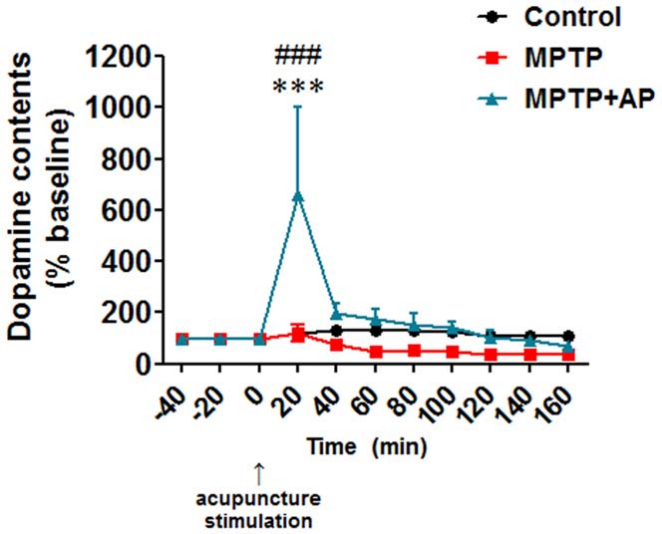
Time courses of dopamine (DA) efflux levels in the striatum. Data presented are mean ± SEM. On the day of the behavioral test (on day 12 after the first saline (Control group) or MPTP (MPTP group and MPTP+AP group) injection), extracellular DA levels in the striatum of Control, MPTP, and MPTP+AP mice were measured after a single acupuncture stimulation using microdialysis-HPLC method. Since MPTP+AP mice already received 11 day's acupuncture treatment before the final acupuncture stimulation, MPTP+AP mice received in total 12 day's acupuncture treatment, whereas Control and MPTP mice received only a single acupuncture treatment. At 20 min after acupuncture stimulation, only MPTP+AP group showed an increase in dopamine efflux. The increase in dopamine efflux was returned to its basal level at 120 min after acupuncture stimulation. Data are normalized to the baseline data of each group. The effect of acupuncture stimulation on dopamine efflux was determined by two-way ANOVA analysis with repeated measures over time followed by Bonferroni's post-hoc test. *** P<0.001 versus Control group and ### P<0.001 versus MPTP group.

Since acupuncture treatment improved motor function (Fig. 2E) but did not restore striatal dopamine level (Fig. 3A), we further investigated whether acupuncture treatment modulates dopamine release in the striatum. Using microdialysis-HPLC method, extracellular dopamine levels in the striatum of Control, MPTP, and MPTP+AP mice were measured after acupuncture stimulation. We found that, only in the striatum of MPTP+AP mice, acupuncture stimulation significantly increased dopamine efflux at time point 20 min (*P*<0.001). Dopamine efflux was six-fold higher in MPTP+AP group than in MPTP group (*P*<0.001, Fig. 4). However, dopamine efflux was not altered both in the striatum of Control and MPTP mice at all time points. These results indicate that acupuncture treatment at GB34 did not restore the MPTP-induced depletion of total striatal dopamine but significantly increased dopamine release in the striatum, which may result in improvement of motor fun`ction in this model of PD.

### Acupuncture treatment at GB34 mitigated MPTP-induced increases in the phosphorylation of DARPP-32 and the expression of FosB

**Figure 5:**
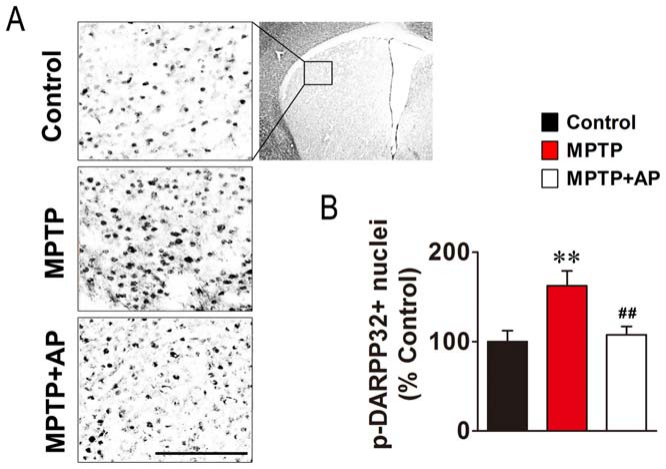
Changes in the phosphorylation state of DARPP-32 by MPTP and acupuncture treatment at GB34. (A) Representative images of immunohistochemical staining for phospho-DARPP-32 at Thr34. (Scale bars: 100 µm.) The top-right panel shows a transverse section of the striatum. The box represents the analysed area. (B) Bar graph of striatal phospho-DARPP-32-positive signal counts (n = 12 per group). Data are normalized to the Control group. MPTP administration induced a significant increase in the phosphorylation of DARPP-32 at Thr34, whereas acupuncture treatment at GB34 significantly mitigated the increased phosphorylation of DARPP-32 at Thr34. **P<0.01 versus Control group and ##*P*<0.01 versus MPTP group via one-way ANOVA followed by a Newman-Keuls test.

Striatal dopamine depletion causes compensatory changes in postsynaptic medium spiny neurons through the hyperactivation of 3′-5′-cyclic adenosine monophosphate (cAMP) signalling [20], which is mediated by dopamine- and cAMP-regulated phosphoprotein of 32 kDa (DARPP-32) [21]. Therefore, we examined MPTP-induced postsynaptic changes using immunohistochemical staining for phospho-DARPP-32 at Thr34. MPTP administration induced a significant increase in the phosphorylation of DARPP-32 at Thr34 in the MPTP group (162.7±16.5% of Control group) in comparison to the Control group (100.0±12.2% of Control group, *P*<0.001). Acupuncture treatment at GB34 significantly mitigated the increased phosphorylation of DARPP-32 at Thr34 in the MPTP+AP group (107.8±9.1% of Control group, *P*<0.001, Figs. 5A, B).

**Figure 6:**
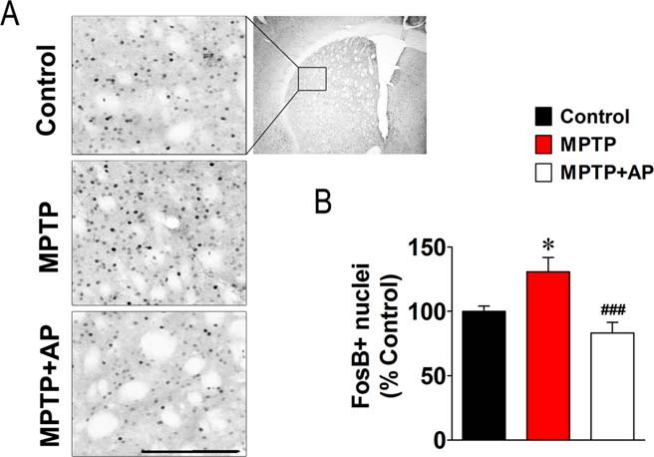
Changes in the expression of FosB by MPTP and acupuncture treatment at GB34. (A) Representative images of immunohistochemical staining for FosB. (Scale bars: 100 µm.) The top-right panel shows a transverse section of the striatum. The box represents the analysed area. (B) Bar graph of striatal FosB-positive nuclei counts (n = 12 per group). Data are normalized to the Control group. MPTP administration increased FosB expression, whereas acupuncture treatment at GB34 reduced the MPTP-induced increase of FosB expression. *P<0.05 versus Control group and ###*P*<0.001 versus MPTP group via one-way ANOVA followed by a Newman-Keuls test.

In addition, we looked for another change in postsynaptic markers of dopamine depletion in the striatum. Because MPTP administration is known to upregulate expression of immediate early gene, FosB [22], [23], [24], we also examined changes of FosB expression in the striatum. Consistent with previous reports, MPTP administration induced a significant increase in FosB expression in the MPTP group (130.7±11.2% of Control group) in comparison to the Control group (100.0±4.1% of Control group, *P*<0.05). Acupuncture treatment at GB34 significantly reduced the MPTP-induced increase of FosB expression in the MPTP+AP group (83.2±8.3% of Control group, *P*<0.001, Figs. 6A, B). These results demonstrate that MPTP-induced increases in the phosphorylation of DARPP-32 and the expression of FosB were mitigated by acupuncture treatment at GB34, suggesting that the acupuncture treatment may normalize MPTP-induced abnormal postsynaptic changes.

## Discussion

The present study demonstrates that acupuncture treatment improved motor function against MPTP most likely through an enhancement of dopamine availability in the synaptic cleft rather than the restoration of the levels of total striatal dopamine.

We found that only acupuncture treatment at GB34, but not at control point significantly improved motor function and prevented dopaminergic neuron degeneration against MPTP (Fig. 2), suggesting that the stimulating a specific point is important to produce the benefits of acupuncture. We also found that acupuncture treatment at GB34 alleviated MPTP-induced motor dysfunction and dopaminergic neuron degeneration, and increased dopamine turnover ratios but did not restore the levels of total striatal dopamine (Figs. 2, 3). It has been suggested that increases in DOPAC/DA and HVA/DA ratios in the striatum reflect an increased metabolism and release of dopamine [25]. Therefore, the observed increases in relative HVA concentrations and increases in DOPAC/DA and HVA/DA ratios following acupuncture treatment in MPTP-intoxicated mice (Fig. 3) suggest that acupuncture may induce an increased rate of dopamine metabolism in the protected dopaminergic neurons with an accompanying increase in dopamine release from dopaminergic terminals. Indeed, we found that acupuncture stimulation significantly increased dopamine release only in MPTP+AP group, but not in Control and MPTP groups (Fig. 4). Some studies displayed that MPTP administration increased the dopamine turnover ratio as a compensatory mechanism with the impairment of motor function [26], [27]. In our study, MPTP group also showed mild increase of turnover ratios, but not statistically different with the Control group. However, the animals with the improvement of motor function after acupuncture enhanced dopamine turnover ratios much more significantly than MPTP group. Recently, we reported that acupuncture can restore MPTP-induced impairment of phosphatidylinositol 3-kinase (PI3K)/Akt cell survival pathway, and PI3K/Akt signalling mediates both acupuncture-induced dopaminergic neuron protection and motor function improvement [6]. Given LY294002, a specific inhibitor of PI3K, concomitantly blocked both acupuncture-induced dopaminergic neuron protection and motor function improvement [6], there seems to be a strong correlation between acupuncture-induced neuroprotection and motor function improvement. Therefore, our previous [6] and the present (Figs. 2, 3, 4) results raise one possibility that the acupuncture-induced dopaminergic neuronal protection may lead to increase in dopamine efflux from the surviving dopaminergic neurons, which in turn may result in improved motor function without necessitating restoration of the striatal dopamine content. There are several studies suggesting that the striatal dopamine content is not necessarily correlated with the improvement of motor function [28], [29], [30]. It is well known that dopamine transporter (DAT) density also plays a role in dopamine transmission by modulating dopamine reuptake. Thus, additionally, we performed the immunohistochemical detection of DAT in the striatum to observe the involvement of DAT. Intriguingly, we observed that there were no significant differences in DAT expression levels between MPTP and MPTP+AP groups (Supplementary Fig. S1), whereas MPTP-induced reduction of TH expression in the adjacent striatum was significantly restored after acupuncture treatment (Fig. 2D), which might result in the increases in DOPAC/DA and HVA/DA ratios in the striatum. However, whether the changes in DAT density participate in the improvement of motor function needs further investigation.

Taken together, our results suggest that acupuncture treatment at GB34 ameliorates MPTP-induced motor dysfunction most likely through an enhancement of dopamine availability in the synaptic cleft rather than the restoration of the levels of total striatal dopamine. Alternatively, the acupuncture-induced protection of dopaminergic neurons shown in our study may result in the maintenance of the functional integrity of the SNr, thereby compensating for striatal dopamine depletion. Because, there are several showing that the substantia nigra pars compacta dopaminergic neurons release dopamine not only from the striatal terminal fibres but also from the dendrite network to the substantia nigra pars reticulata (SNr) [31], [32]. The basal ganglia output structure also may participate in the improvement of motor function induced by acupuncture as Jia et al. suggested [30], [33]. However, to clarify the relationship between our results and above hypothesis needs to be further exploration.

MPTP-induced striatal dopamine depletion induces postsynaptic abnormalities [22], [34]. Therefore, if an acupuncture-induced increase in synaptic dopamine availability (or dopamine neurotransmission) can compensate for the MPTP-induced depletion of absolute dopamine levels in the synaptic cleft, a normalization of MPTP-induced postsynaptic abnormalities may occur. Because MPTP-induced postsynaptic changes are often measured by changes in FosB expression [22], [23], [24] and FosB expression is regulated by DARPP-32 signalling [20], we examined MPTP-induced postsynaptic changes of phospho-DARPP-32 at Thr34 and FosB using immunohistochemistry. Consistent with previous reports [22], [23], [24], MPTP intoxication increased FosB expression in the striatum (Fig. 6). We also found an increase in the phosphorylation of DARPP-32 after MPTP intoxication (Fig. 5). Considering the report that 6-hydroxydopamine did not alter the phosphorylation levels of DARPP-32 [35], our result suggests that MPTP-induced DARPP-32 signalling may differ from 6-OHDA-induced DARPP-32 signalling. More importantly, acupuncture treatment significantly reduced MPTP-induced increases in the phosphorylation of DARPP-32 and the expression of FosB (Figs. 5, 6). These results demonstrate that acupuncture treatment mitigated MPTP-induced abnormal postsynaptic changes, suggesting that acupuncture treatment may increase postsynaptic dopamine neurotransmission and facilitate the normalization of basal ganglia activity.

**Figure 7:**
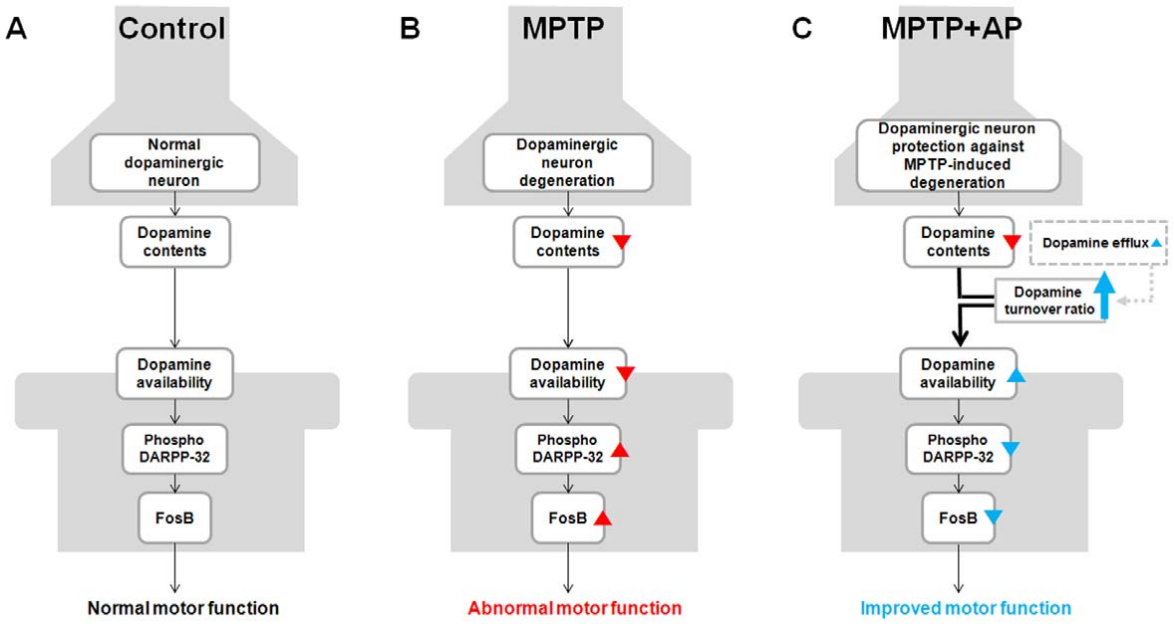
Schematic representation of the effect of acupuncture on dopamine availability and the concomitant postsynaptic response. (A) Normal condition (Control group). (B) MPTP lesioning (MPTP group). (C) Acupuncture treatment at GB34 following MPTP lesioning (MPTP+AP group). Striatal dopamine depletion caused by MPTP-induced dopaminergic denervation leads to abnormally high levels of Thr34-phosphorylated DARPP-32 and the consequent upregulation of FosB in the striatum, which, in turn, may result in abnormal motor function (B). Acupuncture treatment at GB34 following MPTP lesioning alleviated MPTP-induced denervation of dopaminergic neurons but did not restore the levels of total striatal dopamine; however, acupuncture treatment increased dopamine availability likely through increased dopamine release, which, in turn, may lead to the normalization of postsynaptic abnormalities. Therefore, we propose that acupuncture treatment at GB34 improved motor function against MPTP most likely through an enhancement of dopamine availability in the synaptic cleft.

In conclusion, our results suggest that increased dopamine release after acupuncture treatment at GB34 may lead to an enhancement of dopamine availability in the synaptic cleft that in turn, may play an essential role in motor function improvement against MPTP (Fig. 7). From a clinical point of view, one of the major issues in the treatment of PD is how to reduce the adverse effects of L-DOPA. Indeed, various attempts have been made to find a new clinical therapy that can decrease the adverse effects of L-DOPA and increase its efficacy [36]. Our finding that acupuncture improved motor function without necessitating striatal dopamine refilling suggests that acupuncture treatment can be complementary to dopamine replacement therapy. Therefore, we expect that the elucidation of the underlying mechanisms of acupuncture may contribute to the development of novel clinical strategies for the treatment of PD.

## Supporting Information

Figure S1 **Dopamine transporter (DAT) expression in the striatum of each group.** Immunochemistry was performed to detect DAT (1∶1000, Millipore, USA) positive dopaminergic fibers in the striatum. (A) Immunohistochemical staining for DAT-positive dopaminergic fibers in the striatum. (Scale bars: 500 µm.) (B) Bar graph of DAT-positive optical density of fibers in the striatum of each group (n = 7–10 per group). MPTP group showed significant decrease in DAT expression compared to Control, and MPTP+AP group did not alter the decrease. Data are normalized to the Control group. *** *P*<0.001 versus Control group via one-way ANOVA followed by a Newman-Keuls test.
(DOC)

Figure S2 **Nissl staining in the substantia nigra pars compacta.** For Nissl staining, the substantia nigra tissues were stained with 0.5% cresyl violet, and unbiased stereological counts were made. (A) Representative images of Nissl stained neurons in the substantia nigra of each group. Red lines mark the boundaries of the substantia nigra pars compacta (SNc). (B) Bar graph of Nissl stained neuron counts in the SNc of each group (n = 4 per group). Consistent with TH-positive cell counts, a significant recovery of Nissl-positive cell numbers in MPTP+AP group was observed. Data are normalized to the Control group. *P<0.05 versus Control group, and #*P*<0.05 versus MPTP group via one-way ANOVA followed by a Newman-Keuls test.
(DOC)
