# Supplementary figures and images for: Acupuncture Enhances the Synaptic Dopamine Availability to Improve Motor Function in a Mouse Model of Parkinson's Disease
Source: PLoS One. 2011 Nov 22;6(11):e27566. doi: 10.1371/journal.pone.0027566 (PMC3222639; doi:10.1371/journal.pone.0027566)

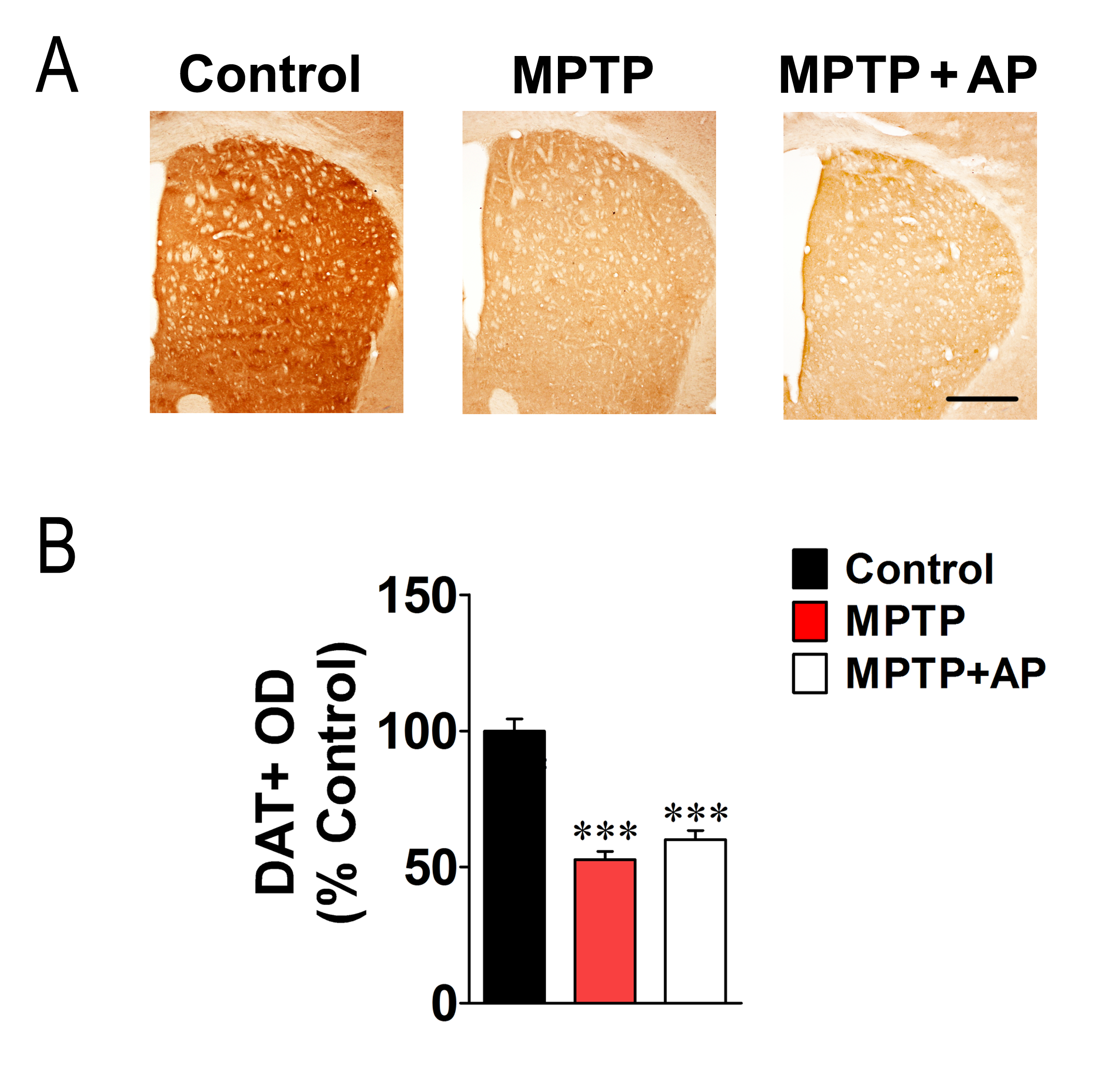

Supplement: Figure S1 — Dopamine transporter (DAT) expression in the striatum of each group. Immunochemistry was performed to detect DAT (1∶1000, Millipore, USA) positive dopaminergic fibers in the striatum. (A) Immunohistochemical staining for DAT-positive dopaminergic fibers in the striatum. (Scale bars: 500 µm.) (B) Bar graph of DAT-positive optical density of fibers in the striatum of each group (n = 7–10 per group). MPTP group showed significant decrease in DAT expression compared to Control, and MPTP+AP group did not alter the decrease. Data are normalized to the Control group. *** P<0.001 versus Control group via one-way ANOVA followed by a Newman-Keuls test. (DOC) [file pone.0027566.s001.doc]

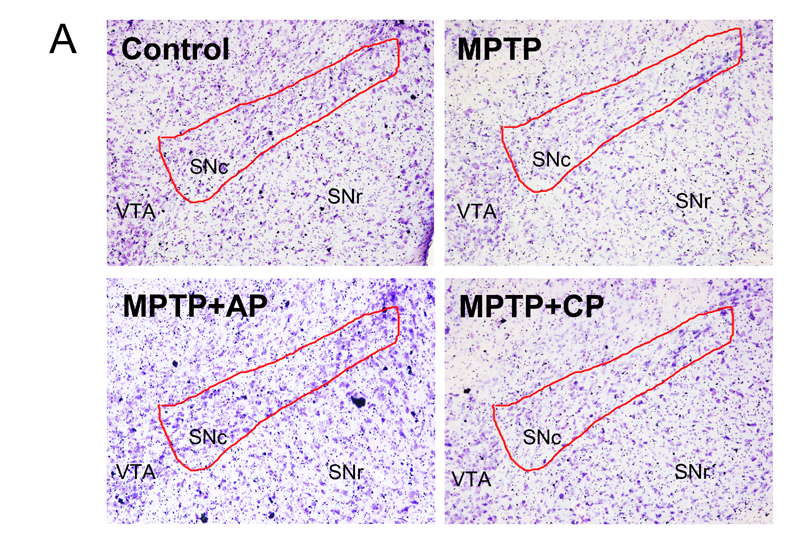

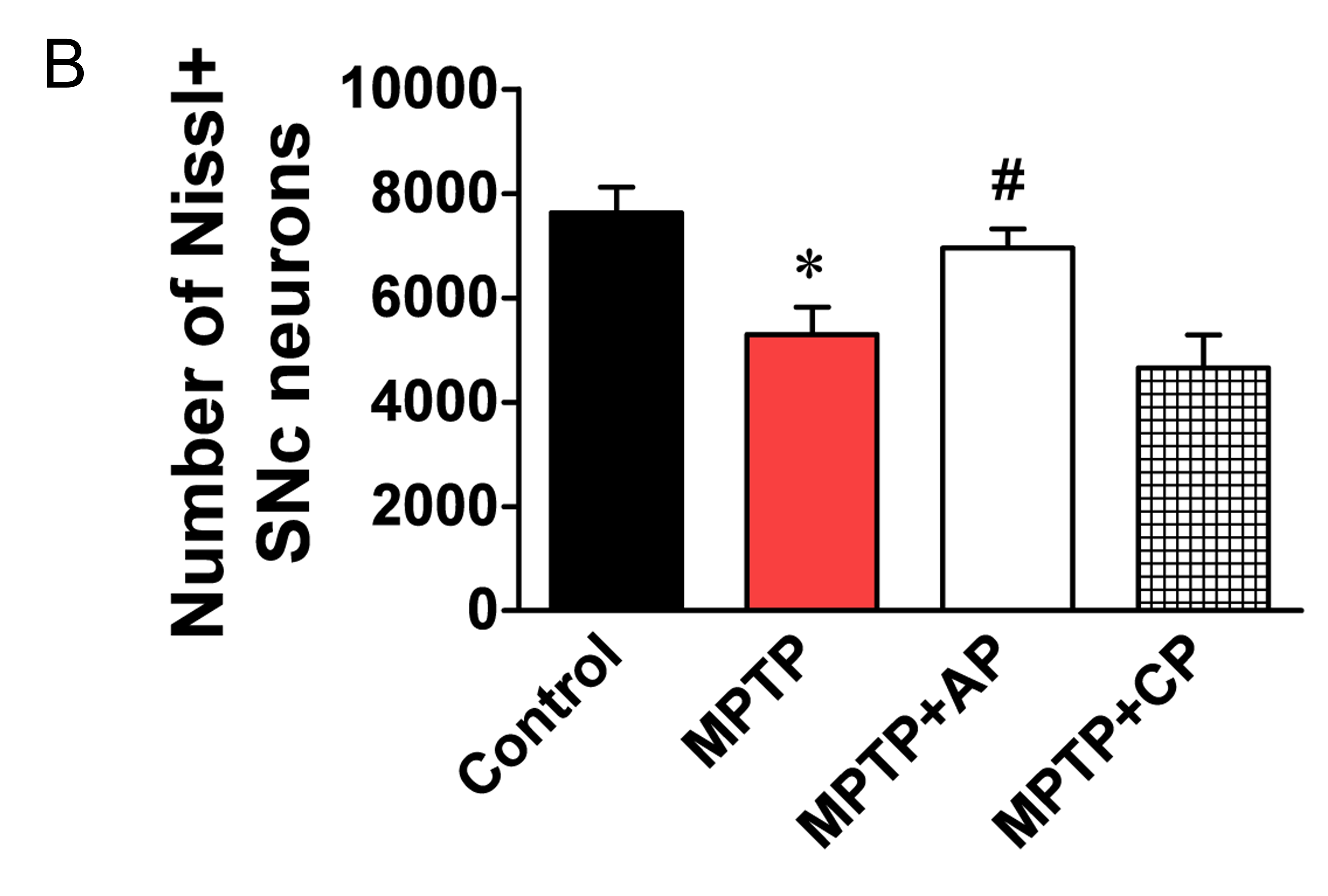

Supplement: Figure S2 — Nissl staining in the substantia nigra pars compacta. For Nissl staining, the substantia nigra tissues were stained with 0.5% cresyl violet, and unbiased stereological counts were made. (A) Representative images of Nissl stained neurons in the substantia nigra of each group. Red lines mark the boundaries of the substantia nigra pars compacta (SNc). (B) Bar graph of Nissl stained neuron counts in the SNc of each group (n = 4 per group). Consistent with TH-positive cell counts, a significant recovery of Nissl-positive cell numbers in MPTP+AP group was observed. Data are normalized to the Control group. *P<0.05 versus Control group, and #P<0.05 versus MPTP group via one-way ANOVA followed by a Newman-Keuls test. (DOC) [file pone.0027566.s002.doc]
